# Ablation of the Chaperone Protein ERdj5 Results in a Sjögren's Syndrome-Like Phenotype in Mice, Consistent With an Upregulated Unfolded Protein Response in Human Patients

**DOI:** 10.3389/fimmu.2019.00506

**Published:** 2019-03-22

**Authors:** Eirini Apostolou, Petros Moustardas, Takao Iwawaki, Athanasios G. Tzioufas, Giannis Spyrou

**Affiliations:** ^1^Department of Pathophysiology, School of Medicine, National and Kapodistrian University of Athens, Athens, Greece; ^2^Academic Joint Rheumatology Program, School of Medicine, National and Kapodistrian University of Athens, Athens, Greece; ^3^Division of Microbiology and Molecular Medicine, Department of Clinical and Experimental Medicine, Linköping University, Linköping, Sweden; ^4^Department of Clinical, Experimental Surgery & Translational Research, Biomedical Research Foundation of the Academy of Athens, Athens, Greece; ^5^Division of Cell Medicine, Department of Life Science, Medical Research Institute, Kanazawa Medical University, Uchinada, Japan

**Keywords:** ERdj5, ER-stress, autoimmunity, salivary gland, Sjögren's syndrome, XBP1, UPR

## Abstract

**Objective:** Sjögren's syndrome (SS) is a chronic autoimmune disorder that affects mainly the exocrine glands. Endoplasmic reticulum (ER) stress proteins have been suggested to participate in autoimmune and inflammatory responses, either acting as autoantigens, or by modulating factors of inflammation. The chaperone protein ERdj5 is an ER-resident disulfide reductase, required for the translocation of misfolded proteins during ER-associated protein degradation. In this study we investigated the role of ERdj5 in the salivary glands (SGs), in association with inflammation and autoimmunity.

**Methods:** *In situ* expression of ERdj5 and XBP1 activation were studied immunohistochemically in minor SG tissues from primary SS patients and non-SS sicca-complaining controls. We used the mouse model of ERdj5 ablation and characterized its features: Histopathological, serological (antinuclear antibodies and cytokine levels), and functional (saliva flow rate).

**Results:** ERdj5 was highly expressed in the minor SGs of SS patients, with stain intensity correlated to inflammatory lesion severity and anti-SSA/Ro positivity. Moreover, SS patients demonstrated higher XBP1 activation within the SGs. Remarkably, ablation of ERdj5 in mice conveyed many of the cardinal features of SS, like spontaneous inflammation in SGs with infiltrating T and B lymphocytes, distinct cytokine signature, excessive cell death, reduced saliva flow, and production of anti-SSA/Ro and anti-SSB/La autoantibodies. Notably, these features were more pronounced in female mice.

**Conclusions:** Our findings suggest a critical connection between the function of the ER chaperone protein ERdj5 and autoimmune inflammatory responses in the SGs and provide evidence for a new, potent animal model of SS.

## Introduction

The Endoplasmic Reticulum (ER) is the site responsible for the proper folding, processing, and trafficking of membrane-bound and secreted proteins. This task is accomplished by a system of ER resident proteins that collectively constitute the ER protein quality control system (ERQC) (1). The ER can respond to stimuli such as accumulation of misfolded proteins and metabolic signals in a condition termed ER-stress, by the activation of the Unfolded Protein Response (UPR) (2). The UPR is an adaptive mechanism comprised of three signaling axes, the IRE1α/XBP1, the PERK/eIF2α, and the ATF6 axis, which are regulated by a common ER resident sensor molecule, BiP. Upon stimulation by excessive misfolded protein load in the ER, IRE1α homodimerizes, and trans-autophosphorylates, causing the alternative splicing of XBP1 to XBP1s, with the latter being a transcription factor that binds directly to stress regulated promoters in the nucleus and controls the expression of many ER-stress related genes. Similarly, activation of the other two branches of the UPR results in the release of the transcription factors CHOP and ATF6p50. Collectively, they promote translation attenuation, ER expansion, enhanced proteolysis and, in unresolved ER-stress cases, inflammation, autophagy, and apoptosis. Deviation from ER homeostasis and activation of the UPR have been implicated in various pathogenic processes of autoimmune diseases, although the mechanism underlying such effects remains largely unknown.

One important aspect linking ER-stress to immune responses is the intersection of UPR and inflammatory signaling, including NF-κB activation, Toll-like receptor signaling and stress kinase signaling (3). UPR signaling plays a role in T cell development and in antigen-triggered B cell differentiation into immunoglobulin producing plasma cells. Moreover, the constitutively active IRE1α/XBP1 axis in mature and immature dendritic cells (DCs) and natural killer (NK) cells is crucial for their development, survival, and function (4). The connection between inflammation and protein misfolding is evident in diseases like TNF receptor-associated periodic syndrome (TRAPS), where mutated, misfolding TNF receptor, and its accumulation in the ER have been linked to the disease (5).

ER-stress and autoimmunity in particular, have been linked through the differential expression of several ER-stress markers. Transcription factors and other markers indicative of ER-stress, such as IRE1α, CHOP, PERK, and XBP1, have been found to be deregulated in systemic lupus erythematosus (SLE) and scleroderma (SSc) (6), XBP1 mutations or deletions in animal models have been associated with inflammatory bowel disease (IBD) (7), and impaired ER-Golgi transport has been associated with lupus nephritis and rheumatoid arthritis (RA) (8).

Primary SS (pSS) is a chronic autoimmune disease characterized by both local exocrinopathy in the salivary gland (SG) and systemic manifestations that affect parenchymal organs. Importantly, 5–10% of SS patients develop B cell non-Hodgkin's lymphoma (9). In human minor SGs (MSGs) from SS patients, pro-inflammatory cytokines have been suggested to perpetuate inflammatory responses and establish a chronic ER-stress state (10), which can lead to autophagy and differential distribution of autoantigens (11). Also, the IRE1α/XBP1 branch of the UPR is differentially regulated in Labial SGs (LSGs) of SS patients, in a manner consistent with chronic ER-stress (12).

ERdj5 is an ER-resident chaperone protein with very potent disulfide reductase and isomerase activity, expressed in secretory cells or following ER-stress (13). ERdj5 has been demonstrated to be a key molecule for the processing of misfolded proteins in the ER. It can bind to BiP with its J domain and enable the reduction of non-native disulfide bonds (14). Among the described functions of ERdj5 are the regulation of ER Ca^+^ homeostasis (15), the facilitation of the correct folding of the LDL receptor (16), the sensitization of neuroblastoma cells to ER-stress-induced apoptosis (17) and the disulfide reduction of misfolded proteins, in order to enable their translocation from the ER to the cytosol during ER-associated protein degradation (ERAD) (18). In the salivary glands in particular, ERdj5 has been shown to contribute in ER protein quality control, since its ablation in mice results in an activated ER-stress response, evident by the upregulation of the alternatively spliced form of XBP1 within the SGs (19).

Given the established interplay between autoimmunity and ER function, and the fact that ERdj5 has an essential role in secretory cells like in the SGs, we aimed to investigate its role in the inflammatory lesions of SGs in the context of pSS. Moreover, we have studied the effects of ERdj5 ablation on the SGs of a mouse model deficient in ERdj5.

## Materials and Methods

### Patients

Thirty female patients with pSS, and 12 non-SS sicca-complaining women (CT) were included in the study. Each patient underwent an MSG biopsy for SS diagnosis. Tissue biopsies of minor salivary gland lobules from patients were obtained from the inner side of the lower lip after local anesthesia. Paraffin sections of these biopsies were used along with acquired blood samples for SS diagnosis. A collection of the remaining sections were used in this study with informed consent from each patient. Patients were diagnosed by the American–European classification criteria (20) and were further categorized according to the grade of MSG inflammatory infiltration in those with mild (SS-I, *n* = 10; Tarpley score: 1, 1–2 focal infiltrates per lobule), intermediate (SS-II, *n* = 9; Tarpley score: 2+, ≥3 focal infiltrates per lobule) and severe lesions (SS-III, *n* = 11; Tarpley score: 3+, diffuse infiltrates associated with severe destruction of acinar glandular structures and loss of tissue architecture) (21). The biopsies from patients whose diagnosis was negative for SS were used as the control group in this study. All but one of those biopsies were totally devoid of inflammatory lesions. In all SS patients, the biopsy focus score (lymphocytic foci/4 mm^2^ of tissue) was ≥1. All non-SS sicca-complaining patients had biopsy focus score < 0.25, while after >5 years, none has had a newer, positive diagnosis.

The exclusion criteria for SS patients were the evidence of lymphoma, sarcoidosis or infection by hepatitis B, hepatitis C or human immunodeficiency virus, and treatment with biological agents before biopsy. The clinical characteristics of the SS patients included in this study are summarized in Table 1. The patient study was approved by the Ethics Committee of the School of Medicine, National University of Athens, Greece (Protocol no. 5107).

**Table 1:** Clinical characteristics of the individuals included in the study.

		**Non-SS sicca controls**	**SS patients**			
	**Features**	**CT (*n* = 12)**	**Total SS (*n* = 30)**	**SS-I patients (*n* = 10)**	**SS-II patients (*n* = 9)**	**SS-III patients (*n* = 11)**
General	Age (years), median (range)	55 (37–74)	55 (18–74)	57 (18–74)	55 (46–70)	52 (32–72)
	No of SS criteria fulfilled, median (range)	1 (1–3)	4 (3–6)	4 (3–5)	4 (3–6)	6 (3–6)
	Duration (years) of sicca symptoms, median (range)	5 (4–6)	3 (0.33–17)	2 (0.66–6)	3 (1–10)	4 (0.33–17)
Histological (MSG biopsy)	Biopsy focus score (number of lymphocytic foci/4 mm^2^), median (range)	0 (0–0.25)	2.35 (1–6.91)	1.18 (1–1.44)	2.4 (1.6–3.75)	5.56 (3–6.91)
	Tarpley biopsy score, median (range)	0 (0–2)	2 (1–4)	1 (1–1)	2 (2–4)	3 (3–4)
Clinical	Arthralgias (%)	50.0%	32.4%	50.0%	44.4%	18.2%
	Arthritis (%)	25.0%	5.6%	10.0%	11.1%	0.0%
	SG enlargement (SGE) (%)	8.3%	24.3%	10.0%	11.1%	63.6%
	Reynaud's phenomenon (%)	8.3%	39.4%	40.0%	44.4%	45.5%
	Parenchymal organ involvement (%)	8.3%	10.0%	10.0%	11.1%	9.1%
	Lung involvement (%)	8.3%	5.4%	10.0%	0.0%	9.1%
	Renal involvement (%)	0.0%	5.0%	0.0%	11.1%	9.1%
	Liver involvement (%)	0.0%	0.0%	0.0%	0.0%	0.0%
	Indicative of vasculitis involvement (%)	0.0%	16.7%	10.0%	0.0%	36.4%
	Palpable purpura (%)	0.0%	10.0%	0.0%	0.0%	36.4%
	Vasculitis (%)	0.0%	2.4%	10.0%	0.0%	0.0%
	Peripheral neuropathy (%)	0.0%	8.6%	10.0%	22.2%	0.0%
	Lymphoma (%)	0.0%	5.1%	0.0%	0.0%	18.2%
Laboratory	Anti-Ro/SSA and/or La/SSB positive (%)	0.0%	63.3%	40.0%	77.8%	72.7%
	Anti-Ro/SSA positive (%)	0.0%	58.1%	40.0%	66.7%	72.7%
	Anti-La/SSB positive (%)	0.0%	32.3%	10.0%	44.4%	45.5%
	Rheumatoid factor positive (%)	16.7%	38.2%	20.0%	33.3%	72.7%
	C3-levels, median (range)	111 (74–171)	95 (63–181)	90 (66.8–181)	113.5 (79–144)	88 (63–98.3)
	C4-levels, median (range)	28.5 (12–43)	23.1 (7.5–47)	36.2 (14–45.6)	23 (13–47)	23 (7.5–41)
	Cryoglobulinemia (%)	0.0%	5.3%	0.0%	0.0%	18.2%
	Hypergammaglobulinemia (%)	0.0%	29.7%	0.0%	44.4%	63.6%
	Leukopenia (%)	0.0%	5.6%	10.0%	11.1%	0.0%
	ESSDAI score	NA	3 (0–9)	1.5 (0–3)	3 (0–5)	5 (0–9)

### Mouse Model

For the experimental evaluation of the role of ERdj5 in SS, we utilized the mouse model of 129SV ERdj5 Knockout mice (ERdj5^−/−^, cohort designation: KO) and their respective 129SV wildtype controls (cohort designation: WT). This mouse model has been produced by the deletion of the exon containing the start codon for ERdj5 translation, producing mice unconditionally deficient in ERdj5 (19). The study cohorts included male or female mice of three age groups, 1.5, 7, and 12 months old (m.o.); 12 experimental groups: Male/Female x 1.5 m.o/7 m.o./12 m.o. x WT/KO. Depending on litter size for each age-matched pair of groups, the sample size was between 7 and 10 animals per animal group. Mice were maintained under monitored, pathogen free conditions in the experimental animal facility of the Biomedical Research Foundation of the Academy of Athens. The housing environment was set at temperature of 22–23°C, 45–55% humidity, a 12:12 h light/dark cycle with lights on at 07:00 a.m. Animals were given free access to drinking water and standard chow diet. The animal experiments were approved by the Animal Care and Use Committee, Veterinarian Administration, Attiki prefecture (Protocol no. K/1279/11).

### Morphometric Analysis of ERdj5 and XBP1s Staining in Human MSGs

The *in situ* expression of ERdj5 and XBP1s in human MSGs was visualized immunohistochemically. In this study, we used four lobules from each patient, all paraffin embedded and sectioned together under the same microscopy slide. For ERdj5, the positive staining and signal intensity in the whole tissue surface and in the secretory ductal epithelium of MSGs were quantitatively measured (see Supplemental Methods). Values were expressed as a percentage of the total tissue area and average stain intensity of positive pixels. For XBP1s, which gave an exclusively nuclear staining, positively stained nuclei were counted in the total tissue area, and expressed as a percentage over the total nuclei count of the tissue. All morphometric image manipulations and measurements were conducted with automated custom-built scripts for Image J (v.1.56j; FIJI distribution) software.

### Histological and Immunofluorescence Examination of Mouse SGs

Tissue samples of whole submandibular salivary glands were obtained from all animal groups. For the morphometric analysis of the inflammatory lesions, lymphocytic infiltrations were defined as aggregates of >50 leukocytes and measured with the use of Image J software on images covering the entire area of the tissue section. Immunofluorescence staining on different sections of the same tissues was performed in order to visualize T cells, B cells, and macrophages (CD3, B220, F4/80, respectively) within the lesions.

### Detection of Antinuclear Antibodies (ANAs) in Murine Serum

Anti-nuclear antibodies were detected in blood sera of mice using the HEp-2 ANA kit (Inova Diagnostics, San Diego, CA, USA). All procedures were performed according to the manufacturer's instructions (see Supplemental Methods). Quantitative measurement of anti-SSA/Ro52, anti-SSA/Ro60, and anti-SSB/La in the murine serum was performed with ELISA assays (Signosis, Santa Clara, CA, USA). All procedures were performed according to the manufacturer's instructions (see Supplemental Methods).

### *In situ* Cell Death Assay

Cell death within murine SGs was evaluated using the TUNEL method (see Supplemental Methods) with the use of the terminal transferase recombinant kit (Roche Diagnostics GmbH) and Alexa Fluor568-conjugated dUTPs (Molecular Probes, MA, USA).

### Salivary Gland Function

Saliva flow was measured on both sexes of KO and WT 12 months old animals, after stimulation by IP injection of pilocarpine solution. The total volume of secretion over time was recorded (see Supplemental Methods) and saliva secretory flow rate was calculated as the volume of saliva per g of mouse weight as a function of time required for each collection.

### Cytokine Levels in Murine Serum and Salivary Glands

Levels of the murine cytokines IFN-γ, IL-1α, IL-2, IL-10, IL-17A, IL-18, IL-23, and TNF-α were measured both in sera and protein lysates of submaxillary glands using the ProcartaPlex Multiplex Immunoassay Magnetic bead panel (Invitrogen, Carlsbad, CA, USA) and a Luminex100 instrument according to the manufacturer's instructions (see Supplemental Methods).

### Statistical Analysis

The unpaired two-tailed non-parametric *t*-test was used for the comparison between experimental groups vs. controls, either in clinical samples or murine experimental data. For the comparison between multiple patient groups of differing lesion severity and multiple animal subgroups of various sexes and ages, we used one-way ANOVA with Tukey's test correction for multiple comparisons. All statistical calculations were performed using the GraphPad Prism v.6.05 software. For all analyses, a *p*-value < 0.05 was considered significant.

## Results

### ERdj5 Is Expressed in Human MSGs and Was Upregulated in MSGs of SS Patients With Severe Inflammatory Lesions

**Figure 1:**
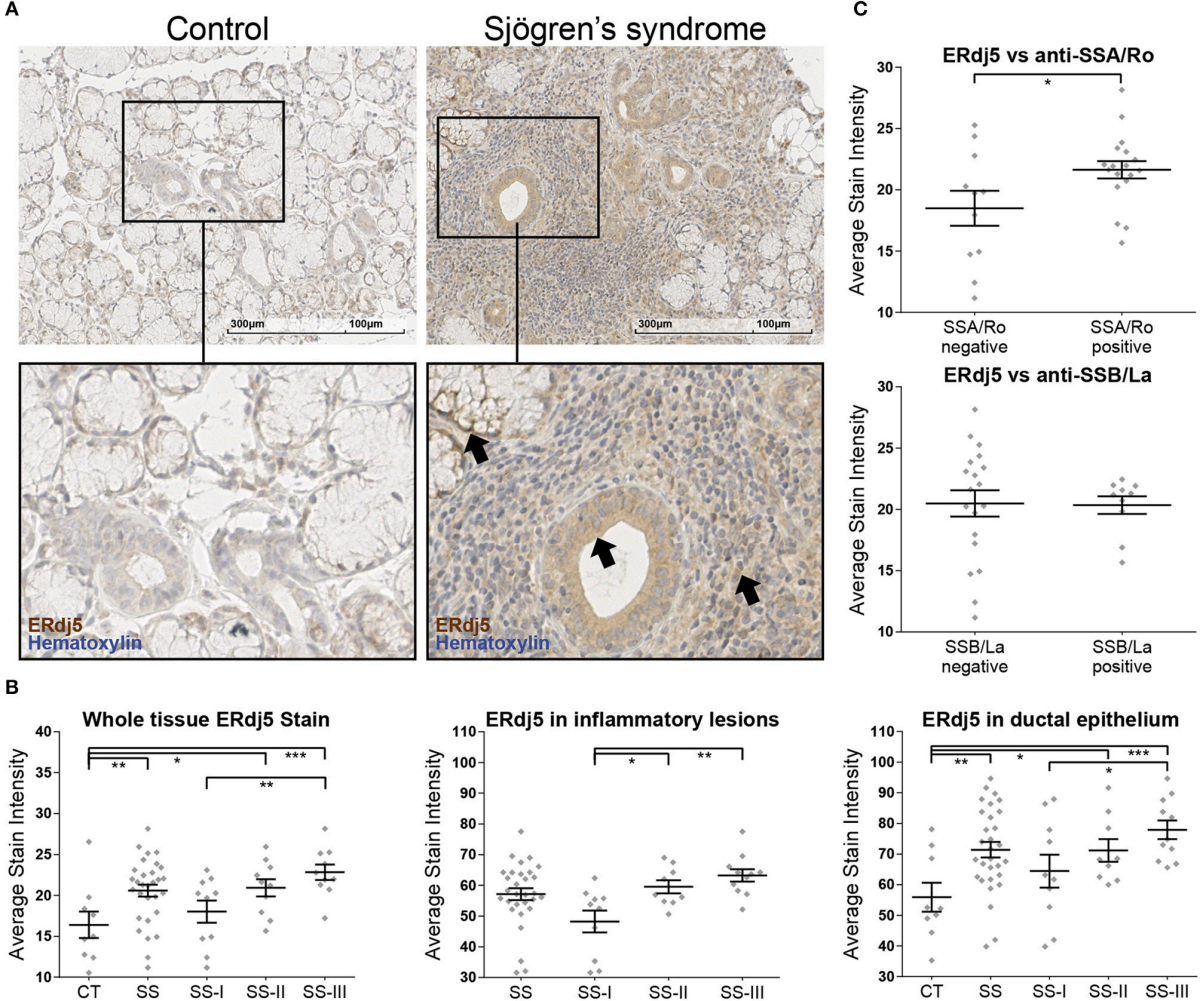
Immunohistochemical detection of ERdj5 in human MSG tissues and morphometric analysis. **(A)** Representative images of ERdj5 staining in CT and SS subjects. Objective magnification: 20x. Positive signal: brown; counterstain: hematoxylin/blue. Arrows: Positive signal for ERdj5 in the acinar cells (left), in the ductal epithelium (middle), and within the inflammatory region (right). **(B)** Morphometric analysis of *in situ* ERdj5. Patients are classified into three SS subgroups by the grade of the inflammatory lesions. Left panel: Stain intensity of positive signal within the entire tissue. Middle panel: Stain intensity of positive signal within the inflammatory lesions. The CT group had no inflammatory lesions to be measured and is omitted from the chart. Right panel: Stain intensity of positive signal within the ductal epithelium. **(C)** Correlation between whole tissue ERdj5 stain intensity and presence of autoantibodies. Upper panel: patients were classified according to serum reactivity to Ro/SSA. Lower panel: patients were classified according to serum reactivity to La/SSB. In all charts, data are represented as individual points for each subject, overlaid by a horizontal mean value ± SEM. Group “SS” encompasses all patients, and was not included in the ANOVA analysis, only in *t*-test vs. CT. Statistically significant differences indicated as ^*^ for *p* < 0.05, ^**^ for *p* < 0.01, and ^***^ for *p* < 0.001.

Immunohistochemical analysis showed that ERdj5 is expressed in the MSGs of both SS patients and non-SS sicca-complaining controls. Positive ERdj5 staining was observed in the ductal and acinar epithelium and in the infiltrating mononuclear cells (MNCs) (Figure 1A). Analysis of the stain intensity within the positively stained areas showed that the signal for ERdj5 was stronger in SS patients (Figure 1B, left panel; CT vs. SS: 16.4 ± 1.6 vs. 20.6 ± 0.7, *t*-test *p* = 0.0125). Comparison between lesion severity subgroups revealed that the ERdj5 positive signal was higher with increasing lesion severity (ANOVA *p* = 0.0052; CT vs. SS *p* = 0.0069, CT vs. SS-II *p* = 0.0152, CT vs. SS-III *p* = 0.0008, SS-I vs. SS-III *p* = 0.0084). This signal was localized within the inflammatory lesions (Figure 1B, middle panel; ANOVA *p* = 0.0011; SS-I vs. SS-II *p* = 0.00173, SS-I vs. SS-III *p* = 0.0014) and the ductal epithelium (Figure 1B, right panel; ANOVA *p* = 0.0071; CT vs. SS *p* = 0.0037, SS-I vs. SS-III *p* = 0.0259). Significant difference in ERdj5 staining intensity also emerged between patients negative or positive for anti-SSA/Ro (Figure 1C, upper panel; Negative anti-SSA/Ro vs. positive anti-SSA/Ro *t*-test *p* = 0.037: 18.49 ± 1.42, *n* = 11 vs. 21.63 ± 0.708, *n* = 18), while no statistically significant difference emerged when patients were classified according to anti-SSB/La serum reactivity (Figure 1C, lower panel).

### Sjögren's Syndrome Patients Have Elevated ER-Stress in the Salivary Glands

**Figure 2:**
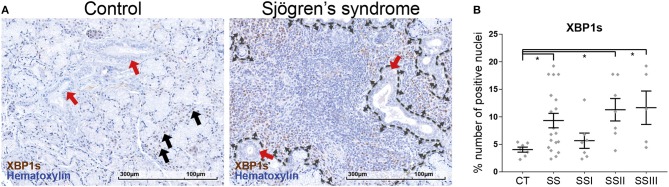
Immunohistochemical detection of XBP1s in human MSG tissues and morphometric analysis. **(A)** Representative images of spliced XBP1 staining in CT and SS subjects. Objective magnification: 20x. Positive signal: brown; counterstain: hematoxylin/blue. Black arrows: Positive nuclei in myoepithelial cells. Red arrows: Ductal epithelium, negative for XBP1s stain. Dashed line traces a large inflammatory lesion, with the small arrows pointing inwards, at the lesion periphery and positive cells. **(B)** Morphometric analysis of the percentage of positively stained for XBP1s nuclei to the total number of nuclei within the tissue sections. Data are represented as individual points for each subject, overlaid by a horizontal mean value ± SEM. Statistically significant differences indicated as ^*^ for *p* < 0.05.

Staining of patient salivary gland biopsy sections for spliced XBP1 (Figure 2) revealed that SS patients have elevated levels of UPR activation, as evident by the higher percentage of nuclei that are positive for XBP1s within the tissue (CT vs. SS *t*-test *p* = 0.025: 4.05 ± 0.45%, *n* = 7 vs. 9.31 ± 1.31%, *n* = 19). Further analyzing the results according to disease severity revealed that the elevated XBP1s positive nuclei count was evident in the SS-II and SS-III groups, with SS-I not presenting a difference compared to CT (ANOVA *p* = 0.0106; CT vs. SS-I *p* = 0.908, CT vs. SS-II *p* = 0.0308, CT vs. SS-III *p* = 0.0402). The positively stained nuclei were distributed within the tissue in a distinct pattern: Myoepithelial cells were sparsely positive in both control subjects and SS patients, with the latter having a higher percentage of positive cells. In the SS group, small inflammatory foci had a homogeneous distribution of positive cells within the lesion. In contrast, the large inflammatory infiltrations had an outer layer of densely positive cells, with the vast majority of the cells in the center negative. Rare mucous and serous cells were positive in SS patients, while the ductal epithelial cells were negative for XBP1s staining (Figure 2A).

### ERdj5^−/−^ Mice Develop Spontaneous Periductal Inflammation in SGs

**Figure 3:**
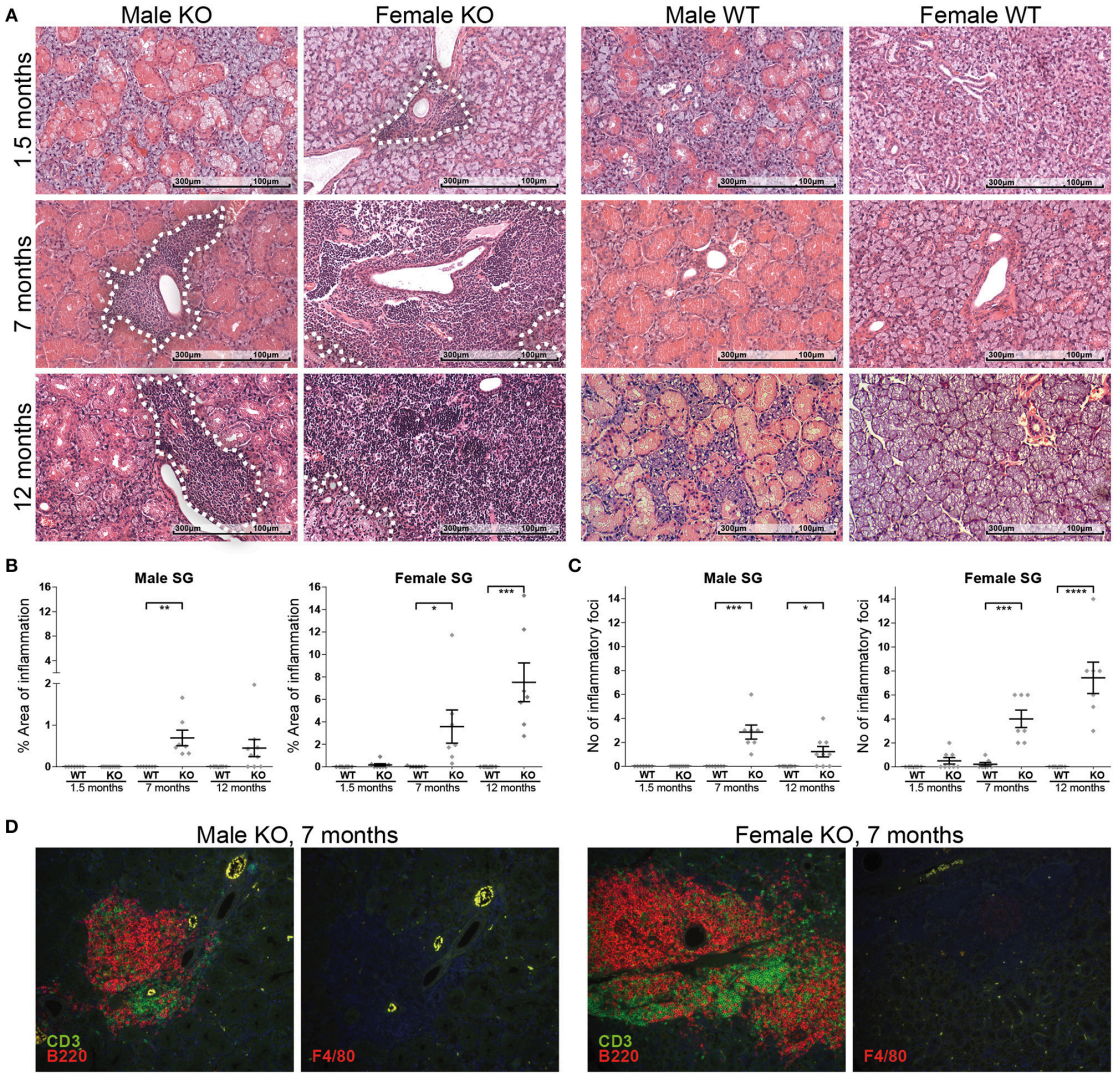
Incidence, progression, and composition of inflammatory lesions in ERdj5^−/−^ mice. **(A)** Histological examination of SG sections from male and female, ERdj5 knockout and wildtype, 1.5, 7, and 12 months old (m.o) animals stained with hematoxylin and eosin (H&E). Inflammatory lesion areas have been marked with a white dashed outline. **(B)** Morphometric measurement of the percentage of tissue surface occupied by inflammatory cells. **(C)** Inflammatory foci count (aggregates of >50 leukocytes) in whole SG tissue area. Data in all charts are represented as individual points for each subject, overlaid by a horizontal mean group value ± SEM, with statistically significant differences indicated as ^*^ for *p* < 0.05, ^**^ for *p* < 0.01,^***^ for *p* < 0.001 and ^****^ for *p* < 0.0001. **(D)** Representative immunofluorescent and double immunofluorescent staining for markers of inflammatory cells (B lymphocytes: B220/red, T lymphocytes: CD3/green, Macrophages: F4/80 / red) in salivary gland tissues from ERdj5^−/−^ male and female at 7 months of age. Sequential sections were stained for the closest localization between multiple stains for each animal. Red/Green-combined yellow color is caused by autofluorescence as indicated in F4/80 stains where no green fluorophore was used. Objective magnification: 20x.

The earliest evidence of inflammation was observed already from 6 weeks (in female KO mice) and the grade and severity of the inflammatory lesions developed over age (Figure 3A), as assessed by the quantitative analysis of inflammation area (Figure 3B; Male panel: 7 m.o. WT vs. KO *t*-test *p* = 0.0032. Female panel: 7 m.o. WT vs. KO *t*-test *p* = 0.0332, 12 m.o WT vs. KO *t*-test *p* = 0.0004) and inflammatory foci (Figure 3C; Male panel: 7 m.o. WT vs. KO *t*-test *p* = 0.0004, 12 m.o. WT vs. KO *t*-test *p* = 0.0183. Female panel: 7 m.o. WT vs. KO *t*-test *p* = 0.0003, 12 m.o. WT vs. KO *t*-test *p* < 0.0001). Interestingly, the observed inflammatory lesions were more prevalent and severe in female KO mice compared to age-matched males. We did not detect any inflammatory lesions in age and sex matched wildtype animals, with the exception of one female 7 month old animal with one small (~50 leucocytes) inflammatory infiltration focus.

The composition of inflammatory infiltrates in KO mice remained unaltered over age. The majority of the cells is constituted mainly by B220+ cells and CD3+ cells in an approximate ratio of 2/1 (Figure 3D). Infiltration of SGs with macrophages and neutrophils was only scarcely observed in inflammatory lesions.

### ERdj5^−/−^ Mice Exhibit Serum Autoantibodies With Higher Titers

**Figure 4:**
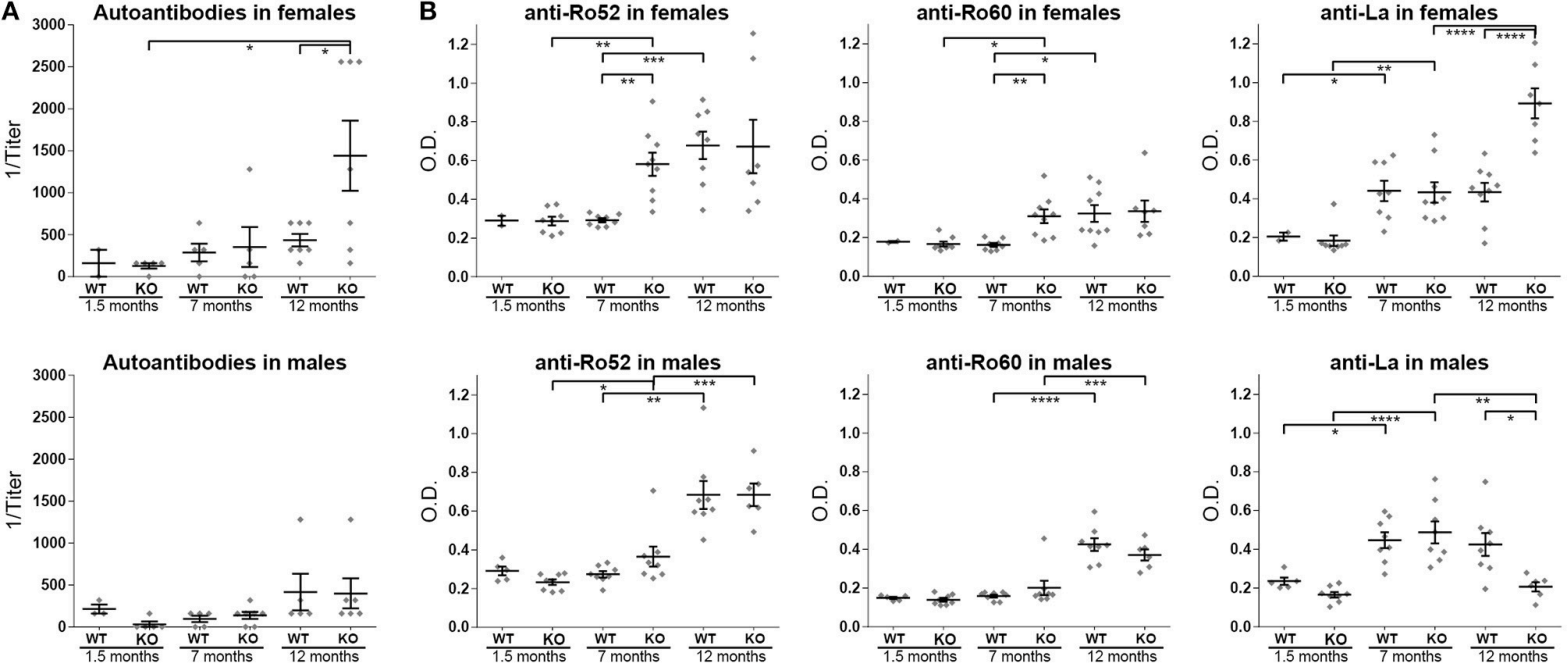
Autoantibodies in the ERdj5^−/−^ animal sera. **(A)** Serum concentrations of anti-nuclear antibodies (ANAs) in female (top panel) and male (bottom panel) ERdj5^−/−^ mice and wildtype controls at 1.5, 7, and 12 months of age. **(B)** Detection of specific autoantibodies in the serum of female (top panels) and male (bottom panels) ERdj5^−/−^ mice and wildtype controls at 1.5, 7, and 12 months of age. Left panels: detection of anti-SSA/Ro52 autoantibodies; Middle panels: detection of anti-SSA/Ro60 autoantibodies; Right panels: detection of anti-SSB/La autoantibodies. Data are represented as individual points for each subject, overlaid by a horizontal mean group value ± SEM. Statistically significant differences are indicated as ^*^ for *p* < 0.05, ^**^ for *p* < 0.01, ^***^ for *p* < 0.001 and ^****^ for *p* < 0.0001.

KO mice produced anti-nuclear antibodies, detected in circulation (Figure 4A and Supplementary Figure 1) and the titers of female KO mice were upregulated over age (12 m.o. WT vs. 12 m.o. KO *t*-test *p* = 0.0354, 12 m.o. KO vs. 1.5 m.o. KO *t*-test *p* = 0.0258). Specific detection of anti-SSA/Ro52, anti-SSA/Ro60 and anti-SSB/La autoantibodies in the serum revealed a significant difference in the kinetics of all three autoantibodies in female ERdj5^−/−^ animals (Figure 4B). Specifically, anti-Ro52 and anti-Ro60 were significantly higher at the age of 7 months compared to age-matched wildtypes (anti-Ro52 female ANOVA *p* = 0.0001: 7 m.o. WT vs. 7 m.o KO *p* = 0.0044; 0.292 ± 0.011, *n* = 8 vs. 0.581 ± 0.059, *n* = 9. Anti-Ro60 female ANOVA *p* = 0.001: 7 m.o. WT vs. 7 m.o KO *p* = 0.0016; 0.162 ± 0.010, *n* = 8 vs. 0.301 ± 0.035, *n* = 9). At 12 months, both of these autoantibodies were also elevated in the wildtype females, with no detectable differences between groups. In contrast, female KO mice did have significantly higher titers of anti-SSB/La autoantibodies at 12 months (anti-La female ANOVA *p* < 0.0001: 12 m.o. WT vs. 12 m.o KO *p* < 0.0001; 0.434 ± 0.048, *n* = 9 vs. 0.892 ± 0.078, *n* = 7). Regarding younger ages, anti-SSB/La autoantibodies were significantly elevated at 7 months compared to 1.5 months regardless of sex or genotype, but there were no detectable differences between WT vs. KO groups of the same age. Interestingly, male mice did not reveal any significant difference at any age regarding anti-SSA/Ro52 and anti-SSA/Ro60, but the 12 months old KO group had significantly lower amounts of anti-SSB/La compared to wildtypes (anti-La male ANOVA *p* < 0.0001: 12 m.o. WT vs. 12 m.o KO *p* = 0.0105; 0.424 ± 0.059, *n* = 8 vs. 0.207 ± 0.024, *n* = 6).

### Cytokine Signature in the Serum and in Submaxillary Glands of ERdj5^−/−^ Mice

**Figure 5:**
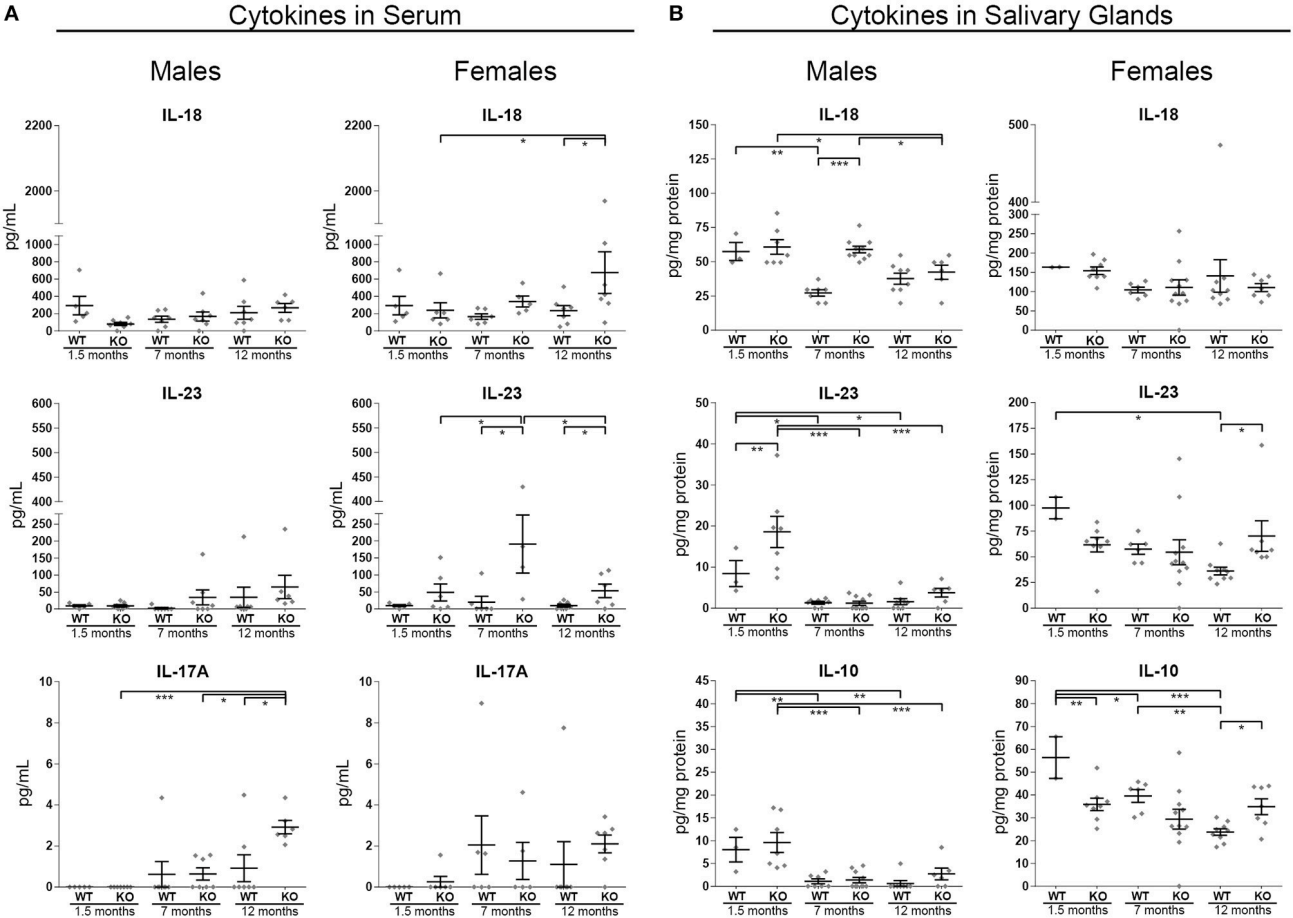
Inflammatory cytokine levels in murine serum and in SG tissue protein extracts from male and female animals of three age groups (1.5, 7, and 12 m.o.). Samples were analyzed with the Multiplex immunoassay magnetic bead technology. **(A)** Quantitative identification of IL-18, IL-23, and IL-17A in the serum. **(B)** Quantitative identification of IL-18, IL-23, and IL-10 in the SG tissue. In all charts, data are represented as individual points for each subject, overlaid by a horizontal mean group value ± SEM, with statistically significant differences indicated as ^*^ for *p* < 0.05, ^**^ for *p* < 0.01 and ^***^ for *p* < 0.001.

Cytokine levels that were found to be deregulated in the blood sera of KO mice are illustrated in Figure 5A (the rest of the examined cytokines, IFN-γ, IL-1α, IL-2, and TNF-α were either at undetectable levels or not differing between groups). IL-18 was significantly higher in the serum of 12 month old female KO animals compared to both their respective controls and to the younger, 1.5 m.o. KO animals (12 m.o. WT vs. KO *p* = 0.0164, 1.5 m.o. KO vs. 12 m.o. KO *p* = 0.0218). IL-23 was found to be significantly upregulated in the female KO group at 7 and 12 months of age, compared to the respective controls (ANOVA *p* = 0.0036; *p* < 0.05 in all comparisons of group 7 m.o. KO with groups 7 m.o. WT, 1.5 m.o. KO, and 12 m.o. KO). IL-17A was upregulated at 12 months of age in male KO animals (ANOVA *p* = 0.008; *p* < 0.02 in all comparisons of group 12 m.o. KO with groups 12 m.o. WT, 1.5 m.o. KO, and 7 m.o. KO).

Within the SGs of male animals, IL-18 was significantly higher in the tissue of 7 m.o. KO mice compared to age matched wildtype animals (ANOVA *p* < 0.0001). Nevertheless, such a result did not emerge in the female groups. In WT animal SGs, IL-23, and IL-10 exhibited a pattern of reducing concentration with age. Interestingly, in the female KO mice this was not the case, intermediate levels of IL-10 and IL-23, were retained throughout all age groups (Figure 5B). As a result, IL-10 was found at significantly lower levels in the KO animals in a young age. For the same reason, in the 12 months old female animals, IL-10 and IL-23 were significantly higher in the KO group, when compared to their age-matched controls (Figure 5B; IL-23 female panel: 12 m.o. WT vs. KO *p* = 0.0183, IL-10 female panel: ANOVA *p* = 0.0004; 1.5 m.o. KO vs. 1.5 m.o. WT *p* = 0.0044, 12 m.o. KO vs. 12 m.o. WT *p* = 0.0143).

### Enhanced Cell Death in the Submaxillary Glands of ERdj5^−/−^ Mice

**Figure 6:**
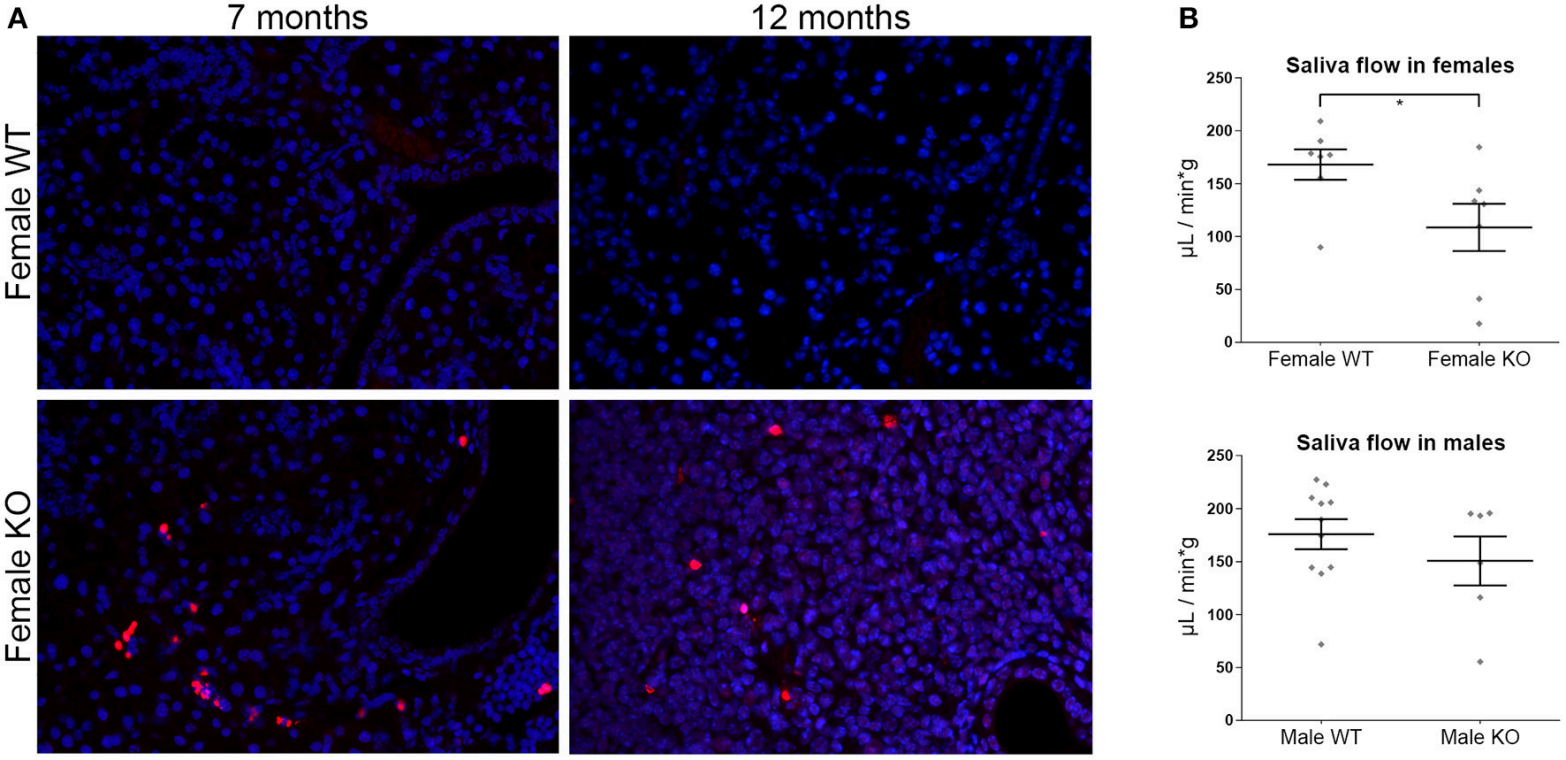
**(A)** TUNEL assay in murine salivary gland tissue sections. Representative images from TUNEL staining (red) in female ERdj5^−/−^ and wildtype animals of 7 and 12 months old. The nuclei were stained with DAPI (blue). ERdj5^−/−^ animals had positive signal within the inflammatory areas. Objective magnification: 20x. **(B)** Functional assay of saliva flow in 12 months old mice. Measurements shown in μL of saliva per minute per g of animal body weight. Data are represented as individual points for each subject, overlaid by a horizontal mean group value ± SEM. The statistically significant difference is indicated as ^*^ for *p* = 0.045.

Within the SGs of KO mice, TUNEL positive material was prominent both in inflammatory infiltrates scattered throughout the lesions and in acinar epithelial cells and was more pronounced in female KO mice (Figure 6A). Experimental murine models suggest that apoptosis constitutes an early event in SS (22), which is consistent with the pronounced effect in female mice as early as at 7 months of age.

### Female ERdj5^−/−^ Mice Have Reduced Salivary Gland Function

We tested the salivary gland function in our experimental model following pilocarpine stimulation. Saliva flow rate was significantly reduced in 12 months old female KO vs. WT (*t*-test *p* = 0.0452), while no significant difference was observed in the male animals of the same age (Figure 6B).

## Discussion

The present study provides evidence for the involvement of the ER Quality Control system (ERQC) in the SGs in the context of Sjögren's syndrome. Specifically, we demonstrate that ERdj5 is involved in the pathophysiology of pSS and that ERdj5^−/−^ mice constitute the first described ER-stress related model of SS, manifesting a plethora of the established traits of the disease.

### ERdj5 Is Involved in the Pathophysiology of SS in Human Patients

As is evident from previous studies, ER-stress can contribute to the pathophysiology of SS. We have previously demonstrated that ERdj5 is a key regulator of the UPR through the PERK axis, inhibiting the phosphorylation of eIF2α by inactivating PERK (17). Another UPR axis involved in LSGs of SS patients is the IRE1α/XBP1 pathway (12). In our study, we have indeed confirmed the upregulation of spliced (activated) XBP1 within the salivary glands of SS patients along with the upregulation of ERdj5, a key ERQC element. The sensor and mediator of these pathways, BiP, has been shown to be selectively expressed in the acinar and ductal epithelial cells in the MSGs (23), while ER-stress induction in a human SG epithelial cell line *in vitro* caused XBP1 mediated autophagy, which was argued to be a protective mechanism against autoantigen re-localization due to apoptotic cell death (11). Moreover, an XBP1 mediated up-regulation of Ro52, which participates in the quality control of IgG1 has been demonstrated in epithelial cells of SS patient LSGs (24). This finding suggests a possible mechanism for the involvement of the ER-stress machinery in autoimmune conditions. Since targets of XBP1s include known SS related autoantigens, it stands to reason that the activation of this branch of the UPR may lead to an increased presence of molecules with autoantigenic potential, their subsequent recognition by the immune system and the establishment and perpetuation of inflammation. Additionally, ER-stress can interfere with antigen presentation, as has been the case in XBP1 mediated miR-346 induction. This microRNA regulates immune responses by targeting genes of the major histocompatibility complex (MHC) class I, interferon-induced genes, and the ER antigen peptide transporter 1 (TAP1), which is necessary for proper MHC class I-associated antigen presentation (25). As it is unclear whether ER-stress precedes or follows the establishment of the SS pathology, it is important to recognize that it can be induced by transcription factors and the action of cytokines involved in other cellular stress events, like oxidative stress and nucleolar stress. The latter may also result in ribosomal and translational stress causing ER-stress in turn. All those stress pathways crosstalk with NFκB signaling, making them relevant to inflammatory responses (26, 27).

The higher ERdj5 signal in SG biopsies from pSS patients highlights its involvement in the pathological manifestations of SS. The predominant expression of ERdj5 in the epithelial compartment of MSGs is consistent with the fact that epithelial cells possess a central role in SS: They are intrinsically activated, expressing inflammatory mediators and specific, traditionally expressed in immune cells, adhesion molecules, and receptors (28), and act both as targets and orchestrators of local autoimmune inflammatory responses, hence the etiological name of SS as “autoimmune epithelitis” (29).

By their nature, chaperone proteins can associate with other proteins, including autoantigens, and can act as recruiting agents in antigen presentation pathways (30). Calreticulin in particular, has been found to participate in antigen presentation of the Ro60 epitopes (31). Additionally, many ER chaperones can be detected in the serum, and some of them have been shown to act as autoantigens themselves, with autoantibodies against BiP (32), HSP47 (33), calreticulin (34), calnexin (35), and GRP94 (34, 35) documented in autoimmune diseases. While ERdj5 could be a new addition to this list of antigenic molecules, the effect of its ablation in mice suggests a more indirect role: The overexpression of ERdj5 in SS patients could be related to a protective mechanism and a compensatory effort of the remaining intact epithelium to produce the necessary proteins. Indeed, the upregulation of the IRE1α/XBP1 branch of the UPR in SS patients, reported in previous studies (12, 24) and confirmed in our experiments, supports this model. Such a connection between ERAD proteins and autoimmunity has been documented in the function of HRD1/synoviolin in synovial cells in RA (36). Also, the inability of the ER to cope with protein misfolding leading to immune reactions is prevalent in HLA-B27 misfolding and augmented production of IL-23 in experimental models of spondyloarthritis (37) as well as in ankylosing spondylitis (38). This could provide a paradigm related to the observed connection between ERdj5 and autoimmunity. Our data indicate ER-stress as the underlying connection that explains the counterintuitive finding of ERdj5 upregulation in patients and the autoimmune responses in mice with ERdj5 ablation. The activation of the IRE1α/XBP1 axis of the UPR in patients and the activation of the same branch of the UPR in the salivary glands of ERdj5^−/−^ mice, that has been demonstrated in previous studies (19), support this idea.

### The ERdj5^−/−^ Mouse: A Potent Animal Model of SS

The pathology that arises in ERdj5^−/−^ mice is induced at an early state and closely resembles multiple facets of the human disease. In this study we have characterized the phenotypic traits of this animal model, which include SG inflammatory infiltrates, serum autoantibodies, reduced saliva secretion, excessive cell death, and deregulated cytokine levels within the SG tissue and in the serum, all with higher severity in females. The importance of a potent animal model for the study of SS is highlighted by the numerous studies that have aimed to develop or describe models of the disease. We believe that ERdj5^−/−^ mice are a valuable addition in the list of available tools for the study of SS, as they are positive for a plethora of features of SS that ideally should be fulfilled by an animal model (39), like few other described models are. The phenotypic characteristics of our experimental model are summarized, along with previously described animal models of SS, in Table 2 (40–49).

**Table 2:** Phenotypic characteristics of ERdj5^−/−^ mice and other mouse SS models.

		**Inflammatory infiltrates in SG**	**SG tissue damage**	**Serum AutoAbs**	**Salivary hypofunction**	**Female predilection**	**Cytokines in serum**	**Cytokines in SGs**	**Parenchymal inflammation**	**Other phenotypes**
Knock-out	ERdj5 KO	+ B-cells, T-cells (2:1 ratio)	+	+ in females anti-Ro anti-La	+	+	Elevated ♀: IL-23, IL-18 ♂: IL-17A	Elevated ♀: IL-23, IL-10	Kidney, occasional infiltrations in liver	nd
	Id3 KO	+ IFN-γ and IL-4 producing lymphocytes	nd	+ Anti-Ro, Anti-La, Autoreactivity to ductal cells	**+**	–	nd	nd	Occasional infiltrations in kidney and lung	Lachrymal gland inflammation and hypofunction. Tumors in numerous organs. Late onset of SS-like phenotype. Skin lesions by over-scratching
	IkB-ζ KO	–	–	+ Anti-Ro, Anti-La	nd	nd	nd	nd	Interstitial pneumonia	Lachrymal gland inflammation, apoptosis, and hypofunction. Periocular dermatitis. Conjunctivitis. Splenomegaly. Lymphadenopathy. Elevated serum IgGs
Transgenic-overexpression	BAFF	+ B-cells predominantly	+	- for Anti–Ro- for Anti-La	+	–	nd	nd	Late onset nephritis. Occasional lung, liver infiltrations	SLE-like. SS-like with age. Reduction in T-regs. Germinal centers in SGs. Hyperactive B-cells
	IL-12 in SJL strain	+	nd	+ ANAs, Anti-La	+	+	nd	nd	Lung inflammation	Lachrymal gland inflammation. Mild autoimmune thyroiditis. Affected osmoregulation of SGs
Chimeric	SS PBMCs in NOD-scid IL-2rγ (null)	+ CD4 predominantly	nd	nd	+	nd	Elevated IFN-γ, IL-10, IL-17, IL-2, IL-6, TNFα	nd	nd	Type-I diabetes. Lachrymal gland inflammation
Spontaneous models	MRL/lpr	+ IFN-γ and IL-17 producing CD4+	nd	+ Anti-Ro, Anti-La	–	– for SG+ for lachrymal	nd	IFN γ, IL-1β, IL-6, TNFα	nd	SLE. Lachrymal gland inflammation. Early death. Aggressive autoimmune lympho-proliferative disorder
	NOD	+ Dynamic CD4+ PD1+ FOXP3-, mainly Th1	+	+ Anti-Ro, Anti-La	+	+ SG in ♀ Lachr. in ♂	IFNs	IL-1β, IL-2, TNFα, IFN-γ, IL-10, IL-17, IL-12(p40)	Lung inflammatory infiltrations. Kidney antibody deposition	IDDM. Thyroiditis-like. SLE. Myasthenia. SS phenotype affected by housing conditions. Weak association with HLA haplotypes
	NOD Aec1. Aec2	+ Progressive T-cell and B-cell infiltrations	+	+	+	+	Differentially expressed ± IFNs	± IFNs. Elevated cytokines	nd	Anti-M3R antibodies. Lachrymal gland hypofunction in females
	NZB/NZW F1	Augmented by incomplete Freund's adjuvant IP injection (F.adj. inj.)	nd	ANAs and anti-Ro augmented by F.adj.IP	Augmented by F.adj. and polyI:C injection	+	nd	Augmented by polyI:C injection	Kidney	SLE-like phenotype. Non-specific inflammatory stimuli. Conjunctivitis
Immunization	Ro60 in BALB/c	+	nd	nd	+	nd	nd	nd	nd	Anti-Ro, Anti La in saliva. Late onset. Requires multiple immunizations
	Alum in NZM2758	+	nd	+ ANAs	+	nd	nd	“*Inflammasome components”*	nd	“*P2X7 receptor-dependent phenotypes”*

*+, SS trait is reported in the relevant literature; –, SS trait reported negative in the relevant literature; nd, not determined in the relevant literature; italics, trait discussed as possibility but not shown in experimental data*.

The spontaneous development of inflammatory infiltrations involved mostly epithelial sites. This development of periductal inflammation in SGs of ERdj5^−/−^ mice was reminiscent of inflammatory lesions in MSGs of SS patients, consisting mainly of B and T lymphocytes. In our experimental model, like in previously described murine models of SS, the inflammatory infiltrates evolve in size over age. The cellular composition of those infiltrates remained similar though at all time-points. Lesions in the SGs were accompanied by higher titers of ANAs systemically, with the titer of ANAs being positively correlated to the extent of the inflammation and the number of lesion foci. Autoantibodies are a defining and diagnostic trait of SS pathology, present in many previously described models of the disease. Our mouse model exhibited higher levels of anti-SSA/Ro52 and antiSSA/Ro60 antibodies in females at the age of 7 months, and higher levels of anti-SSB/La antibodies in females at 12 months of age, providing clues for a possible trend that is not easily discernable in human patients who are usually diagnosed when the disease is already established: High anti-SSA/Ro52+60 antibodies might be a trait of early disease stages, while the anti-SSB/La antibodies may have delayed elevation with respect to disease development. Furthermore, even in wildtype animals, these autoantibodies increase naturally with age, so the age choice of an experimental setup may mean the difference between a negative or a positive result regarding these markers. Interestingly, males did not differ at any age point regarding anti-SSA/Ro52+60 autoantibodies, while at 12 months of age we observed reduced anti-SSB/La autoantibodies. More experimentation is required for the interpretation of this result, which could be due to masking by anti-idiotypic antibodies as has been reported to occur specifically for anti-SSB/La antibodies (50), complex formation, and serum depletion, or other regulatory mechanisms.

Chronic inflammation and ensuing tissue damage in SS patients eventually leads to compromised SG function and xerostomia. Reduced salivary function was confirmed in female ERdj5^−/−^ mice, evident by the decreased production of saliva, adding to the traits of this model that are relevant to SS. Importantly, this effect was noted in the experimental group with the most extensive inflammatory lesions, higher number of inflammatory foci, higher titers of ANAs, and anti-SSB/La antibodies in the blood serum. In patients, SS can be accompanied by parenchymal organ involvement, in the form of autoimmune epithelitis (29). Accordingly, limited individuals in our study developed small inflammatory lesions in the liver, and aged ERdj5^−/−^ animals had a marked inflammation in the renal pelvis (not shown). An interesting observation is the sex related difference in the severity of the SS-like phenotype in ERdj5^−/−^ animals, which is more pronounced in female mice. In humans, SS has a strong predilection for women (9:1 female to male ratio) (48). This makes the ERdj5^−/−^ mouse an appropriate model for the study of the human disease.

In ERdj5^−/−^ mice, similarly to SS patients (51), deregulated expression of cytokines has been identified both systemically and within the SGs. IL-23, which, synergistically with IL-18, induces additional proinflammatory cytokines (28) was found in higher levels both in the serum and the SGs of female 12 month old animals, a result consistent with findings in SS patients (52). The higher IL-23 levels were accompanied by serum IL-18 upregulation in the experimental group with the most prominent inflammation and the highest ANA and anti-SSB/La titer, the 12 months old female knockouts. Indeed, in SS, IL-18 has been associated with tissue pathogenesis and the presence of anti-SSA/Ro and anti-SSB/La antibodies (53). In the young KO females, IL-10 was lower in the SG compared to age-matched WT females, while this trend reversed in aged female animals. This finding can be attributed to IL-10s' documented dual role. At early stages, lower levels of IL-10 fail to achieve its anti-inflammatory action. Inversely, IL-10 has been documented to act pro-inflammatory in the presence IFN-α (54), and thus its higher levels in aged female animals could contribute to the lesion progression. The fact that elevated IL-10 levels in SS patients have been correlated with disease severity (51) is consistent with our data, since patients are examined when the disease is already established.

The high occurrence of cell death observed within the severe inflammatory lesions of ERdj5^−/−^ mice agrees with previous clinical (55) and experimental studies (43) and can provide a plausible mechanism for the exposure of autoreactive material to the extracellular space that drives the initiation and the perpetuation of the inflammatory process. Autoantibodies from sera of SS patients are known to be able to activate apoptotic pathways (56). Consistently, our mouse model presented with elevated ANAs and anti-SSB/La antibodies in the groups with increased cell death. Supporting evidence come also from the upregulation of the caspase-induced cytokine IL-18 in the serum of female ERdj5^−/−^ mice, which is found elevated in the saliva of SS models and human SS MSGs (53).

## Conclusions

In summary, ERdj5, as part of the ER quality control machinery, may be considered a key player in maintaining the integrity of the salivary gland, a tissue that is prone to damage, which leads to the detrimental effects of initiation and perpetuation of autoimmune responses. Even though the triggering mechanism(s) are yet unknown, deregulated ER-stress may contribute to the cascades that lead to tissue destruction, antigen presentation and sustained chronic inflammation, making it harder for the tissue to heal itself, ensure integrity and evade triggering autoimmune responses.

## Data Availability

The raw data supporting the conclusions of this manuscript will be made available by the authors, without undue reservation, to any qualified researcher.

## Author Contributions

EA and PM contributed equally in this work. They mutually designed and performed all the experiments and authored the manuscript. TI created the ERdj5 knockout mouse model and critically reviewed the manuscript. AT and GS contributed equally, supervising the design and execution of all experimental procedures, critically reviewing and proofing the manuscript.

### Conflict of Interest Statement

AT has received research grants from Novartis, Pfizer, UCB, AbbVie and GSK pharmaceutical companies, through the National and Kapodistrian University of Athens, outside the submitted work. The funder played no role in the study design, the collection, analysis or interpretation of data, the writing of this paper or the decision to submit it for publication. The remaining authors declare that the research was conducted in the absence of any commercial or financial relationships that could be construed as a potential conflict of interest.
