# Supplementary material for: Ablation of the Chaperone Protein ERdj5 Results in a Sjögren's Syndrome-Like Phenotype in Mice, Consistent With an Upregulated Unfolded Protein Response in Human Patients
Source: Front Immunol. 2019 Mar 22;10:506. doi: 10.3389/fimmu.2019.00506 (PMC6438897; doi:10.3389/fimmu.2019.00506)
Supplement: Supplementary file 1 [file Data_Sheet_1.PDF]

Supplementary Material

Ablation of the Chaperone Protein ERdj5 Results in a Sjögren's Syndrome-Like Phenotype in Mice, Consistent with an Upregulated Unfolded Protein Response in Human Patients.

Eirini Apostolou[#], Petros Moustardas[#], Takao Iwawaki, Athanasios G. Tzioufas[*], Giannis Spyrou[*]

# These two authors contributed equally to this work

*** Correspondence:**
Equally contributing corresponding authors
Giannis Spyrou: giannis.spyrou@liu.se
Athanasions G Tzioufas: agtzi@med.uoa.gr

1 Supplementary Methods

1.1 Clinical and experimental samples

Tissue samples of whole submandibular salivary glands from mice were obtained after the experimental animals were sacrificed by blood draining via the inferior vena cava under ketamine/xylazine intraperitoneal anesthesia (anesthetic solution of 20mg/mL ketamine, 1.33mg/mL xylazine in H_2O; 170mg ketamine and 11.33mg xylazine per kg of animal weight). The acquired blood samples were centrifuged at 900 x g for 10 minutes and the sera were collected and stored at -80ºC for subsequent analysis. For the human samples, biopsies of minor salivary gland lobules from patients were obtained from the inner side of the lower lip after local anesthesia. Tissues were washed in PBS pH=7.4 and fixed in 10% formalin solution (4% formaldehyde, 1% methanol) at 4ºC overnight. After fixation, tissues were washed in tap water, dehydrated in ascending concentrations of ethanol (70%, 80%, 95%, 2x 100%), cleared twice in xylene and embedded – molded into blocks of paraffin wax. Paraffin blocks were sectioned at 5μm thickness (human biopsies) and 3μm thickness (murine samples) in a Leica RM2125 RTS microtome.

1.2 Immunohistochemical staining of human MSGs

ERdj5 and XBP-1s staining of human MSG biopsies was performed using a standard technique using the EnVision system (Dako). Antigen retrieval was performed by microwaving in 10mM Tris, 1mM ethylenediamine tetra-acetic acid (EDTA), pH 9.0. Non-immune fetal bovine serum (10%) and 0.5% H_2O_2 in methanol were used to block non-specific antibody binding and endogenous peroxidase activity, respectively. Permeabilization was performed by 0.1% TritonX-100 in blocking buffer. Staining with primary antibody was performed overnight in a humidified chamber at 4ºC. Negative control staining was performed by replacing primary with irrelevant isotype-matched antibody. After DAB development, sections were counterstained with Harris hematoxylin solution, washed in tap water, dehydrated, mounted with DPX and coversliped.

1.3 Morphometric analysis of ERdj5 staining in human MSGs

At least 10 fields of MSG biopsy images per study subject (at least four glands per section) were acquired by a bright-field color 5Mpixel digital image-acquisition camera connected to a Carl-Zeiss microscope under an 20x objective lens. Using an automated custom built script for the Image J (v.1.56j; FIJI distribution) software, acquired images where color deconvoluted for the isolation of the positive immunohistochemical stain, and the resulting images were processed for the measurement of the positive stain area and stain intensity, both in all the tissue area as well as within the isolated inflammatory lesion and ductal epithelium areas. Positively stained area was expressed as a percentage of the whole tissue area with staining intensity above a universally defined threshold value, while stain intensity was the calculated result of the function "maximum 8-bit pixel brightness (255) – the average pixel brightness of the area of interest".

1.4 Morphometric analysis of XBP1s staining in human MSGs

The total tissue area from one biopsy sample section per each patient was photographed segmentally by a bright-field color 2.3Mpixel digital image-acquisition camera (Sony Pregius 1/1.2'' exmor CMOS) connected to an Olympus microscope under an 20x objective lens. Acquired images where color deconvoluted for the isolation of the positive immunohistochemical stain, and the resulting images were processed for counting the amount of nuclei visible in the brown (DAB) and in the blue (hematoxylin) channel with an automated custom built script for Image J (v.1.56j; FIJI distribution) software.

1.5 Histological examination of mouse salivary glands

Paraffin-embedded sections of salivary glands from the mice of all experimental groups were deparaffinized by immersing in xylene, followed by dehydration in descending concentrations of ethanol (2x 100%, 95%, 80%, 50%) and finally immersion in tap water. The prepared tissue sections were stained with Harris hematoxylin and eosin (H&E) (1min immersion in hematoxylin stain, wash with tap water x3, 1min immersion in Eosin stain), dehydrated in ascending concentrations of ethanol and xylene (80%, 95%, 2x 100% EtOH – 2x xylene) and mounted with DPX . Stained sections were observed at 200x magnification in an inverted microscope.

1.6 Immunofluorescent staining for inflammatory cell infiltrates in mouse SGs

Paraffin-embedded tissues of the mouse salivary glands were sectioned and mounted onto microscope slides. Slides were deparaffinized and dehydrated. Sections were incubated one hour with blocking solution containing 10% FBS diluted in PBS. Sections were incubated with primary antibodies overnight at 4°C. The slides were washed three times with PBS-T, followed by a one-hour incubation with appropriate secondary fluorescent antibodies. The slides were washed thoroughly with PBS-T, treated with Vectashield DAPI (4′,6-diamidino-2-phenylindole)-mounting medium (Vector Laboratories, Burlingame, CA, USA) and overlaid with glass coverslips. Stained sections were visualized at 200x magnification on a Zeiss fluorescent microscope.

1.7 Antibodies and dilutions for Immunohistochemisrty and Immunofluorescence

Anti-ERdj5: mouse monoclonal anti-human/mouse/rat ERdj5 antibody [clone 3C4] (H00054431-M01) from Abnova (Antibodies-online, Atlanta, GA, USA), diluted 1:200 in antibody diluent (Dako, Carpinteria, CA, USA)
Anti-XBP1s: rabbit monoclonal anti-human spliced XBP1 antibody [Clone D2C1F] (product #12782) from Cell Signaling technology (Danvers, MA, USA), diluted 1:100 in PBS supplemented with 2%FBS, 0.2%BSA, 0.05% Triton-X 100.
Ant-CD3: rabbit monoclonal anti-CD3 antibody [clone: SP7] (ab16669) from Abcam (Cambridge, UK) diluted 1:100 in antibody diluent.
Anti-B220: rat monoclonal anti-mouse CD45R/B220 antibody [clone: RA3-6B2] (BD553084) from BD Biosciences (San Jose, CA, USA) diluted 1:2000 in antibody diluent.
Anti-F4/80: rat monoclonal anti-mouse F4/80 antibody [clone Cl:A3-1] (MCA497GA) from Bio-Rad (Oxford, UK) diluted 1:50 in antibody diluent.
Anti-rabbit secondary fluorescent: goat polyclonal anti-rabbit IgG (H+L) antibody Cross-Adsorbed with Alexa Fluor 488 (A-11070) from Life Technologies (Grand Island, NY, USA) diluted 1:200 in antibody diluent.
Anti-rat secondary fluorescent: donkey polyclonal anti-rat Gamma IG Heavy and Light chains antibody conjugated with Alexa Fluor 594 (A-21209) from Invitrogen (Carlsbad, CA, USA) diluted 1:200 in antibody diluent.

1.8 ELISA assays for autoimmunity related autoantibodies in murine serum

Mouse anti-SSA/Ro52, anti-SSA/Ro60 and anti-SSB/La antibodies were quantitatively measured in the serum of all animals using ELISA 96 well plate assays from Signosis, Inc (Santa Clara, CA, USA):
EA-5202: Mouse Anti-SSA (Ro-60) ELISA Kit
EA-5203: Mouse Anti-SSA (Ro-52) ELISA Kit
EA-5204: Mouse Anti-SSB (La) ELISA Kit
For all three assays, sera were diluted 1:150 (2μL serum in 298μL assay diluent) and 100μL of diluted sample were used per assay well. All procedures were conducted according to manufacturer's instructions (direct application of each sample in the appropriate well without prior well soaking, incubation for 60min, aspiration and 3 washes with 1x wash buffer, incubation with 100μl of diluted anti-mouse IgG antibody for 30min, aspiration and 3 washes with 1x wash buffer, development with 100μl of substrate solution for 7 minutes and stop reaction with 50μL stop solution. Optical density (O.D.) was measured in an ELISA plate spectrophotometer at 450nm.

1.9 ProcartaPlex Multiplex Immunoassay Magnetic bead cytokine assay in serum and SG tissue extracts

The serum assay was conducted using 25μL of serum sample and 25μL universal assay buffer per well. For tissue extracts, a piece of approximately 20 mg of frozen tissue was cut from the lower tip of each frozen SG sample and homogenized in RIPA buffer (10 mM Tris-HCl pH 8.0, 1mM EDTA, 1% Triton X-100, 0.1% sodium deoxycholate, 0.1% SDS, 140mM NaCl) supplemented with 1mM PMSF (1:100 from 100mM stock solution) and 1x protease inhibitors solution (1:10 from 10x stock solution). Total protein concentration in the tissue extracts was measured using the Pierce BCA protein assay kit (Thermo Scientific, Waltham, MA, USA) and samples were normalized to 5mg/mL with the appropriate dilution using RIPA buffer. Then, 100mg of total protein (20μL sample) were mixed with 30μL universal assay buffer to be used as samples in the panel. Measurements were exported from the

Luminex 100 IS software to the MasterPlex 2010 software for subsequent analysis and quantification using 5 parameter logistic calibration curves constructed from the provided standard.

1.10 Detection of antinuclear antibodies (ANA) in murine serum

Mouse sera were diluted 1:80 in PBS and incubated on HEp-2-fixed substrate slides for one hour at room temperature in a humidified chamber. After three 5-min washes with PBS, the substrate slides were treated with a 1:200 dilution of AF 555 goat anti-mouse IgG H&L (Life Technologies, Grand Island, NY, USA) in 10% FCS PBS for 1hr at room temperature. After three washes, slides were treated with Vectashield DAPI mounting medium (Vector Laboratories) and overlaid with glass coverslips. Fluorescence was detected by fluorescence microscopy at 400x magnification by using a Zeiss fluorescent microscope.

1.11 TUNEL assay

Sections were de-paraffinized, rehydrated and subsequently incubated at RT for 10 min with the (equilibration) mixture as a blocking step in a humidified chamber. Then, sections were incubated with the TUNEL reaction mixture (terminal transferase recombinant kit -Roche Diagnostics GmbH- and Alexa Fluor 568-conjugated dUTPs -Molecular Probes, MA USA) for 1 hour at 37°C. Reaction was terminated in 2xSSC solution for 15 min at RT. Sections were washed in 1xPBS and counterstained with DAPI for localization of nuclei. As a negative control, sections were treated with reaction mix devoid of the TdT enzyme whereas as a positive control sections were pretreated with DNase (Promega Corporation, WI, USA).

1.12 Salivary gland function

For each animal (KO and WT adult sex and age-matched, 12 months old littermates), 20 pieces of Whatman filter paper 2mm×25mm were marked at 7mm from one end and placed in a hermetically capped 1.5mL microcentrifuge tube. The total weight of the tubes with paper was measured using a precision balance with ±100μg accuracy. Mice were administered an anesthetic solution (20mg/mL ketamine, 1.33mg/mL xylazine in H_2O; 90mg ketamine and 6mg xylazine per kg of animal weight) by intraperitoneal injection, weighed and positioned in a vertex position facing up. Subsequently, they received a low dose of pilocarpine (70μg/mL solution; dosage 0.4μg/g of animal body weight) by intraperitoneal injection. A piece of filter paper was then inserted into the cavity of the right cheek of each mouse until the 7mm mark was level with the two ventral incisors and left in place up to 3 min or until it became visibly wet up to the 7mm mark in order to absorb any saliva secreted. Each piece of filter paper was removed, immediately placed and sealed in its pre-weighed vial and replaced with another. The time of the pilocarpine injection was taken as time zero, and the time in minutes from this point was recorded as each sample was collected. This process was repeated for a total duration of 30 minutes or until the 20 pieces were used. Each vial was re-measured and the weights of all samples were recorded. The difference in total weight of vial plus paper measured before and after collecting saliva was taken as the net weight of total saliva secreted during the collection period. Saliva secretion volume was calculated assuming 1mg = 1μl saliva and then normalized by the weight of the mouse in grams.

2 Supplementary Figures

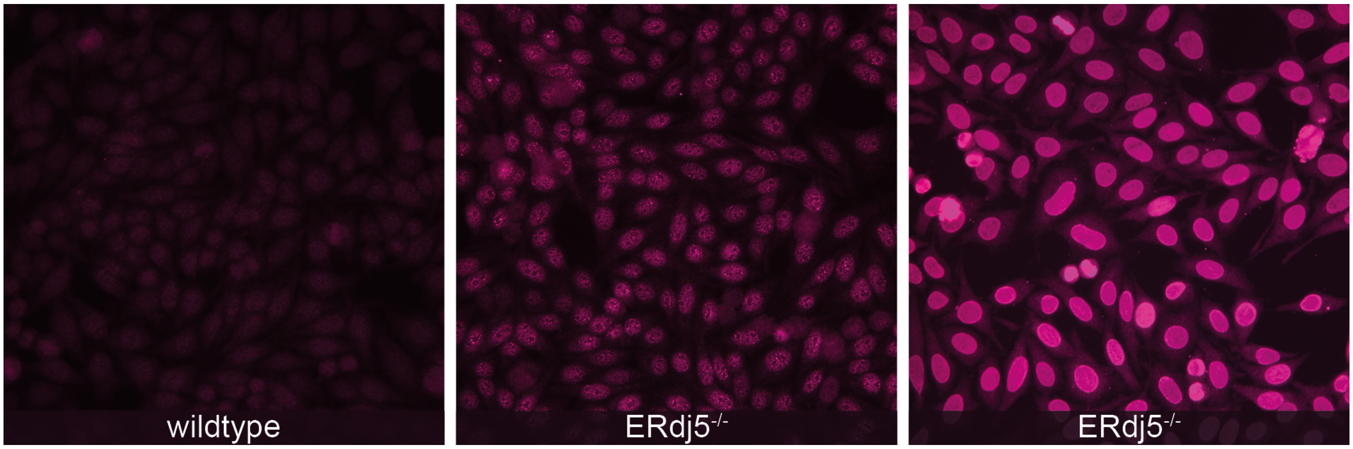

Supplementary Figure 1. Representative images of anti-nuclear antibody (ANA) test with murine serum in the HEp-2 ANA kit under fluorescence. Original magnification: 40x.
